# Scalable high *Q*-factor Fano resonance from air-mode photonic crystal nanobeam cavity

**DOI:** 10.1515/nanoph-2023-0170

**Published:** 2023-06-13

**Authors:** Fujun Sun, Zhihua Li, Bo Tang, Bin Li, Peng Zhang, Ruonan Liu, Gang Yang, Kai Huang, Zhe Han, Jun Luo, Wenwu Wang, Yan Yang

**Affiliations:** Institute of Microelectronics, Chinese Academy of Sciences, Beijing 100029, China; Beijing University of Posts and Telecommunications, Beijing 100876, China

**Keywords:** Fano resonance, integrated photonics, nanobeam cavity, scalable

## Abstract

Fano resonance from photonic crystal nanobeam cavity (PCNC) is important building block for large-scale photonic integrated circuits (PICs) to enable photonic switches and sensors with superior characteristics. Nevertheless, most state-of-the-art demonstrations rely on electron beam lithography (EBL) and operate in dielectric mode. Hence, we theoretically, numerically and experimentally present the characteristics of Fano resonance from optical interference between the discrete state of air-mode PCNC and the continuum mode of side-coupled line-defect waveguide with partially transmitting element (PTE) using deep ultraviolet (DUV) lithography for the first time. Experimentally high average *Q*-factor of ∼1.58 × 10^4^ is achieved for 30 measured devices, which indicates the feasibility of mass manufacture of high-*Q* Fano resonance from air-mode PTE-PCNC. Additionally, the thermo-optic bi-stability and thermal tuning characterizations of the proposed device are discussed. This work will contribute to building ultra-compact lab-on-chip resonance-based photonic components.

## 1 Introduction

In silicon photonics, micro-resonators including micro rings/disks and photonic crystal cavities are important building blocks for large-scale photonic integrated circuits (PICs). Among them, photonic crystal nanobeam cavities (PCNCs) have attracted extensive attention for the investigation of optical sensing [1–6], switching [7, 8], filtering [9, 10], modulating [11–13], and lasing [14, 15], owing to ultra-high quality factor (*Q-*factor), small mode volume (*V*), less free spectrum range (FSR) limitation, and compact footprint. Compared with the usual symmetric Lorentzian resonance line-shape achieved by ordinary side-coupled PCNC structure [2–4, 7, 10, 12, 15], Fano resonance arising from the interference between a discrete mode and a continuum mode has steeper asymmetric shape [16–29]. For the waveguide and PCNC coupling system, it is convenient to realize Fano resonances by intentionally incorporating partially transmitting element (PTE) into the waveguide generating continuum mode [16–24]. The PTE in the waveguide is realized by etching one or several holes, which is used to control and tune the amplitude of the continuum path. Yet, to date, although Fano resonances in PCNCs bear significant potentials towards above mentioned applications such as sensing, switching and so on, they have not been explored thoroughly.

On one hand, the demanding nature of photonic crystal devices in terms of fabrication capabilities is high. The high lithography resolution such as e-beam lithography (EBL) is required to define the small feature size, which is not practical for manufacturing PICs with large number of components. Therefore, recently several groups worked on the development of dielectric-mode PCNCs [30–32] and 2D photonic crystal devices [33–36] defined using deep ultraviolet (DUV) lithography. However, the feasibility of air-mode PCNC and asymmetric Fano resonance from PCNC have not been explored yet. Compared with dielectric-mode PCNC, air-mode PCNC confines large electric fields in low refractive index region, which are suggested for the application of refractive index sensing to achieve higher sensitivity. On the other hand, most reported Fano resonances in PTE-PCNCs are based on the dielectric-modes. Only few Fano resonances from air-mode PTE-PCNCs are reported theoretically and numerically in previous published works, suffering from large insertion loss and low *Q-*factor [37–39]. Therefore, it is desirable to explore the characteristics of Fano resonance from air-mode PTE-PCNC fabricated with DUV lithography.

In this work, Fano resonances from air-mode PTE-PCNCs are exhibited theoretically, numerically and experimentally. Two-hole-assisted PTE is placed in the center of the arcuated side-coupled waveguide. And the air-mode PCNC is formed by quadratically tapering the lattice space of the circular holes from the center to both ends while other parameters remain unchanged. The temporal coupled-mode theory (TCMT) and three dimensional finite-difference time-domain (3D-FDTD) method are utilized to analyze the designed structure, the Fano resonance line-shapes and the corresponding partial *Q*-factors. Experimental results show that the PTE-PCNC produces sharper and higher extinction ratio (ER) Fano line-shape compared to the established Lorentzian resonance structure. Experimentally average total *Q*-factor of ∼1.58 × 10^4^ is achieved for 30 measured resonances. Additionally, the nonlinear and thermal tuning characterizations of the proposed PTE-PCNC device are discussed. The evidence of bi-stability can be clearly seen at input powers over 0.36 mW. And the temperature tuning sensitivity is ∼55.5 pm/°C. To the best of our knowledge, this is the first demonstration of Fano resonance from side-coupled air-mode PCNC in silicon fabricated with DUV lithography. This study will contribute to building ultra-compact lab-on-chip resonance-based components in PICs such as sensors, switches, filters, reflectors, and so on.

## 2 Design and theory

Aiming to fabricate the device on a standard full-process complementary metal-oxide-semiconductor (CMOS) passive multi-project-wafer (MPW) run, the device should be designed to comply with the rules defined for this process. The devices are fabricated by 248 nm-*λ* DUV lithography on SOI wafer with a 3 µm-thick buried oxide (BOX) layer and a 220 nm-thick top-silicon (Si) layer. The corresponding minimum feature size is 180 nm. The silicon layer can be defined by 220 nm-thick full etch and 70 nm-thick shallow etch. And as part of an MPW run, 2 µm-thick silicon oxide (SiO_2_) top-cladding is also included in the process flow. Therefore, for our studied devices, the etched holes are entirely filled with SiO_2_.

### 2.1 Structure design

**Figure 1:**
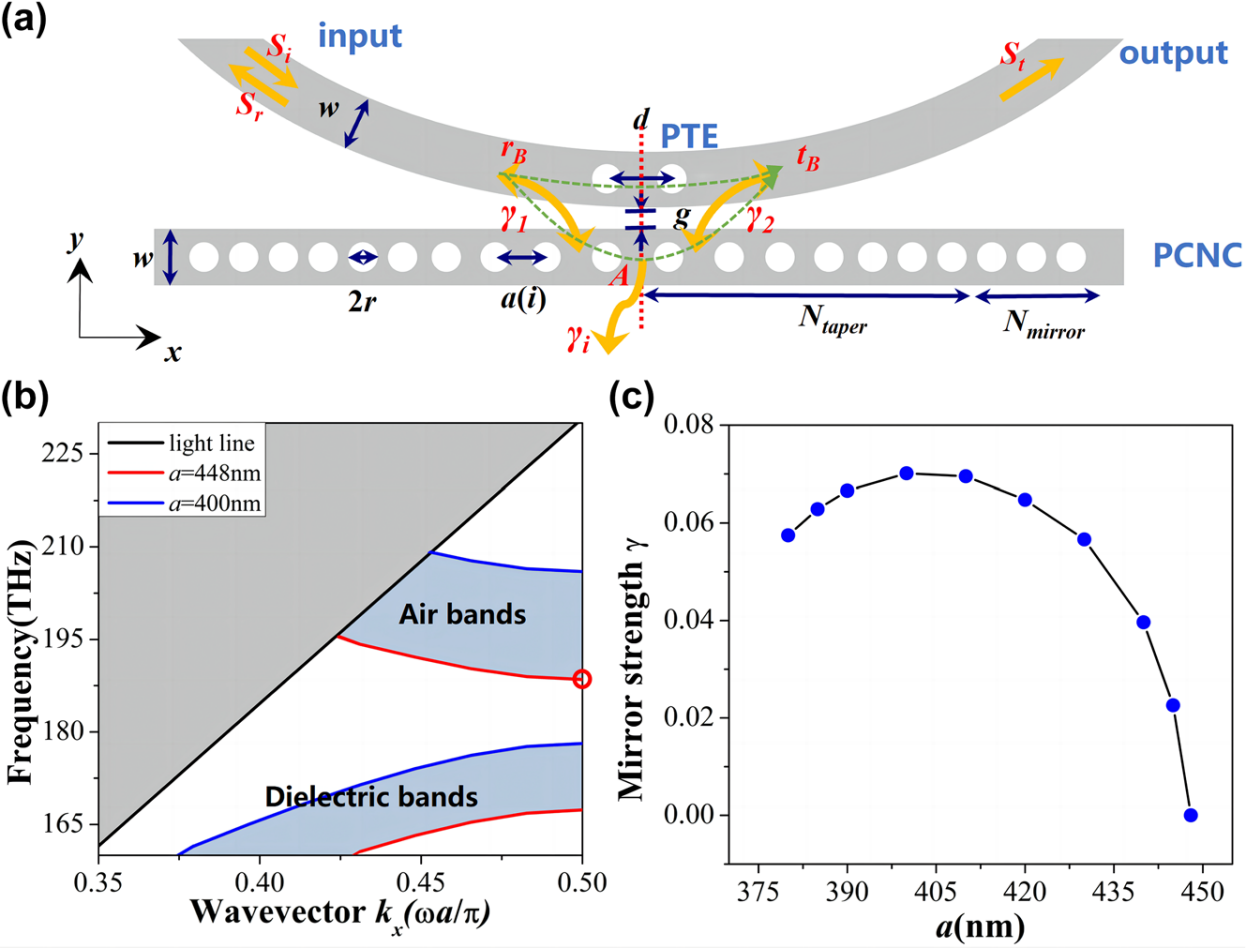
Structure design. (a) Schematics of the proposed air mode Fano resonance PTE-PCNC and side-coupling model. The structure is symmetric with respect to its center (red dashed line). *w* is the width of the waveguide. The air holes have radius *r*, and are kept constant. The lattice constant *a*(*i*) are quadratically modulated from center to both sides. (b) TE dispersion diagram of the PCNC with *a* = 448 nm (red line) and *a* = 400 nm (blue line). The red circle indicates the target resonant frequency. (c) Mirror strengths at different lattice constant from the 3D band diagram simulation.

Figure 1(a) shows the schematic of our proposed Fano resonance device, consisting of a side-coupled bus waveguide with PTE and an air-mode PCNC. Since the fundamental mode profile of the PCNC is highly concentrated in the central region of PCNC, an arc bus waveguide is designed to suppress the coupling of higher order modes. The radius of the arc feeding waveguide is 10 μm. The PTE is simply realized by etching two holes with radius *r* on the bus waveguide. The two holes are symmetric with respect to the red dashed line shown in Figure 1(a). Here, the center-to-center distance between the two air holes in *x* direction is labeled as *d*. For our fabricated devices, the distance *d* is set as 448 nm, which is the same as the lattice constant of the central tapered segment in PCNC. Note that to keep the fabrication uniformity, the holes’ radii are same for PCNC and PTE. As depicted schematically in Figure 1(a), Fano resonance occurs from optical interference between the discrete state (fundamental air-mode) of the PCNC and the continuum mode of the side-coupled line-defect waveguide with PTE. Part of the incident light launched at the input waveguide port with field amplitude *S*_*i*_ passes through the PTE directly, while the other part is coupled to the cavity mode in PCNC. *A* is the field amplitude of the fundamental mode in PCNC with a resonant frequency of *ω*_0_. The energy in PCNC can decay into input and output waveguides with decay rates *γ*_1_ and *γ*_2_, respectively, or decay as an intrinsic loss into the free space with intrinsic loss rate *γ*_*i*_. *t*_*B*_ and *r*_*B*_ are the transmission and reflection coefficients of PTE, respectively, satisfying $t_{B}^{2}+r_{B}^{2}=1$ . The value *t*_*B*_ can be tuned by controlling the distance between the two holes formed PTE, which can be used to engineer the parity and the shape of the Fano resonance spectrum [21–23, 40–42]. *S*_r_ is the reflected field amplitude at the input waveguide port. And the light is coupled out of the structure through outport of the waveguide with the transmitted field amplitude *S*_t_.

The PCNC consists an array of circular holes etched into a Si strip waveguide with a width *w* of 450 nm and a thickness of 220 nm. The diameter of the holes is 2*r* = 180 nm, which conforms the minimum feature size. The structure is symmetric with respect to the red dashed line shown in Figure 1(a). The PCNC is optimized using the deterministic high-*Q* design method introduced by Quan et al. [43]. To create a Gaussian mirror reducing the intrinsic loss, for the tapered region, the hole lattice constant is quadratically tapered from *a*(1) = 448 nm in the center to *a*(*N*_taper_) = 400 nm on both sides (*a*(*i*) = *a*(1) − (*i* − 1)^2^(*a*(1) − *a*(*N*_taper_))/(*N*_taper_ − 1)^2^, *i* increases from 1 to *N*_taper_), while other parameters of the structure remain unchanged. The transverse-electric (TE) band diagram with lattice constant 448 nm and 400 nm simulated by a 3D-FDTD method with Bloch boundary conditions is given in Figure 1(b). Here, *a*(*N*_taper_) is chosen to realize maximum mirror strength (*γ*), as shown in Figure 1(c). The mirror strength *γ* for different lattice constant is calculated by $\sqrt{{\left(w_{2}-w_{1}\right)}^{2}/{\left(w_{2}+w_{1}\right)}^{2}-{\left(w_{0}-w_{m}\right)}^{2}/w_{m}^{2}}$ , where *w*_0_ is the proposed target resonance, and *w*_2_, *w*_1_, and *w*_*m*_ are the air band edge, dielectric band edge, and midgap frequency of each segment, respectively [43]. The air band frequency indicated by red circle in Figure 1(b) is the target resonant frequency. The decrease of lattice constant makes the effective refractive index of the guiding structure decreases, thus the cutoff frequency becomes higher correspondingly, as shown in Figure 1(b).

### 2.2 Temporal coupled-mode theory

Here, we analyze the coupled fundamental air-mode of PTE-PCNC according to temporal coupled-mode theory (TCMT). For an input light with field amplitude *S*_*i*_ = e^−i*ωt*^, therefore, in the steady state, the resonant field amplitude of fundamental mode d*A*/d*t* = −i*ωA*. The equations for the evolution of the PTE-PCNC mode amplitude in time are given by

(1) $$S_{t}=-\text{i}t_{B}S_{i}+\sqrt{2\gamma_{2}}{\text{e}}^{\text{i}\theta_{2}}A$$

(2) $$\frac{\text{d}A}{dt}=-\left(\text{i}\omega_{0}+\gamma_{1}+\gamma_{2}+\gamma_{i}\right)A+\sqrt{2\gamma_{1}}{\text{e}}^{\text{i}\theta_{1}}S_{i}=-i\omega A$$

Where ${\text{e}}^{\text{i}\theta_{1}}$ and ${\text{e}}^{\text{i}\theta_{2}}$ are the phase coupling coefficients of the coupling between the input/out ports and the PCNC, respectively. Accordingly, the power transmission of the PTE-PCNC coupling system can be calculated as follows:

(3) $$T\left(\omega \right)={\left|\frac{S_{t}}{S_{i}}\right|}^{2}={\left|-it_{B}+\frac{2\sqrt{\gamma_{1}\gamma_{2}}{\text{e}}^{\text{i}\left(\theta_{1}+\theta_{2}\right)}}{\gamma_{1}+\gamma_{2}+\gamma_{i}+\text{i}\left(\omega_{0}-\omega \right)}\right|}^{2}$$

The phase term ${\text{e}}^{\text{i}\theta_{1}}$ and ${\text{e}}^{\text{i}\theta_{2}}$ can be calculated from the values of *t*_*B*_, *γ*_1,_ and *γ*_2_ using the requirements of energy conservation and time-reversal symmetry as

(4) $${\text{e}}^{\text{i}\left(\theta_{1}+\theta_{2}\right)}=\text{i}t_{B}\sqrt{\gamma_{2}/\gamma_{1}}\left[\frac{1}{r_{B}{\text{e}}^{-\text{i}P2\theta_{1}}+1}\right]$$

Here, the factor *P* takes the value +1(−1) for the red (blue) parity Fano resonances. The parity of a Fano line is defined by whether the transmission minimum is red- or blue-shifted relative to the maximum [23]. For our proposed symmetric PTE-PCNC design, the fundamental air-mode is odd with respect to the mirror symmetry plane. Therefore, *γ*_1_ = *γ*_2_, *θ*_1_ = *θ*_2_, and $\cos\left(2\theta_{1}\right)=\left(\gamma_{2}-\gamma_{1}\right)t_{B}^{2}-2\gamma_{1}\gamma_{B}^{2}/2\gamma_{1}\gamma_{B}=-\gamma_{B}$ [29]. Correspondingly, for *P* = ±1, Eq. (3) can be deduced as:

(5) $$T\left(\omega \right)={\left|\frac{S_{t}}{S_{i}}\right|}^{2}={\left|-it_{B}+\frac{2\gamma_{1}\left(\text{i}t_{B}\mp \sqrt{1-t_{B}^{2}}\right)}{2\gamma_{1}+\gamma_{i}+i\left(\omega_{0}-\omega \right)}\right|}^{2}$$

Apparently, the cavity loss is composed of the intrinsic loss (characterized by *Q*_*i*_ = *ω*_0_/2*γ*_*i*_) and the coupling loss (characterized by *Q*_*c*_ = *ω*_0_/2(*γ*_1_ + *γ*_2_) = *ω*_0_/4*γ*_1_) to the coupling waveguide. Thus, the net dimensionless total decay rate 1/*Q*_*t*_ can be written as the sum of two decay rates: 1/*Q*_*t*_ = 1/*Q*_*i*_ + 1/*Q*_*c*_. In practice, the intrinsic loss of the cavity includes the radiation loss into the free space (characterized by *Q*_*r*_) and scattering loss induced by fabrication imperfection (characterized by *Q*_*s*_). While, in simulation, only the *Q*_*r*_ is considered. And the *Q*_c_ and *Q*_*r*_ are evaluated by *Q* = *w*_0_ *U*/P using 3D-FDTD simulation, where *ω*_0_ is the resonance frequency, *U* is the electromagnetic energy in the cavity and *P* is the rate of energy loss. Hence, according to Eq. (5), for given *t*_*B*_, *ω*_0_, *Q*_*c*_ and *Q*_*r*_, the transmission spectrum can be calculated. Similarly, for given measured transmission spectrum, *Q*_*c*_ and *Q*_*i*_ can be extracted by fitting to Eq. (5).

## 3 Simulation and optimization

**Figure 2:**
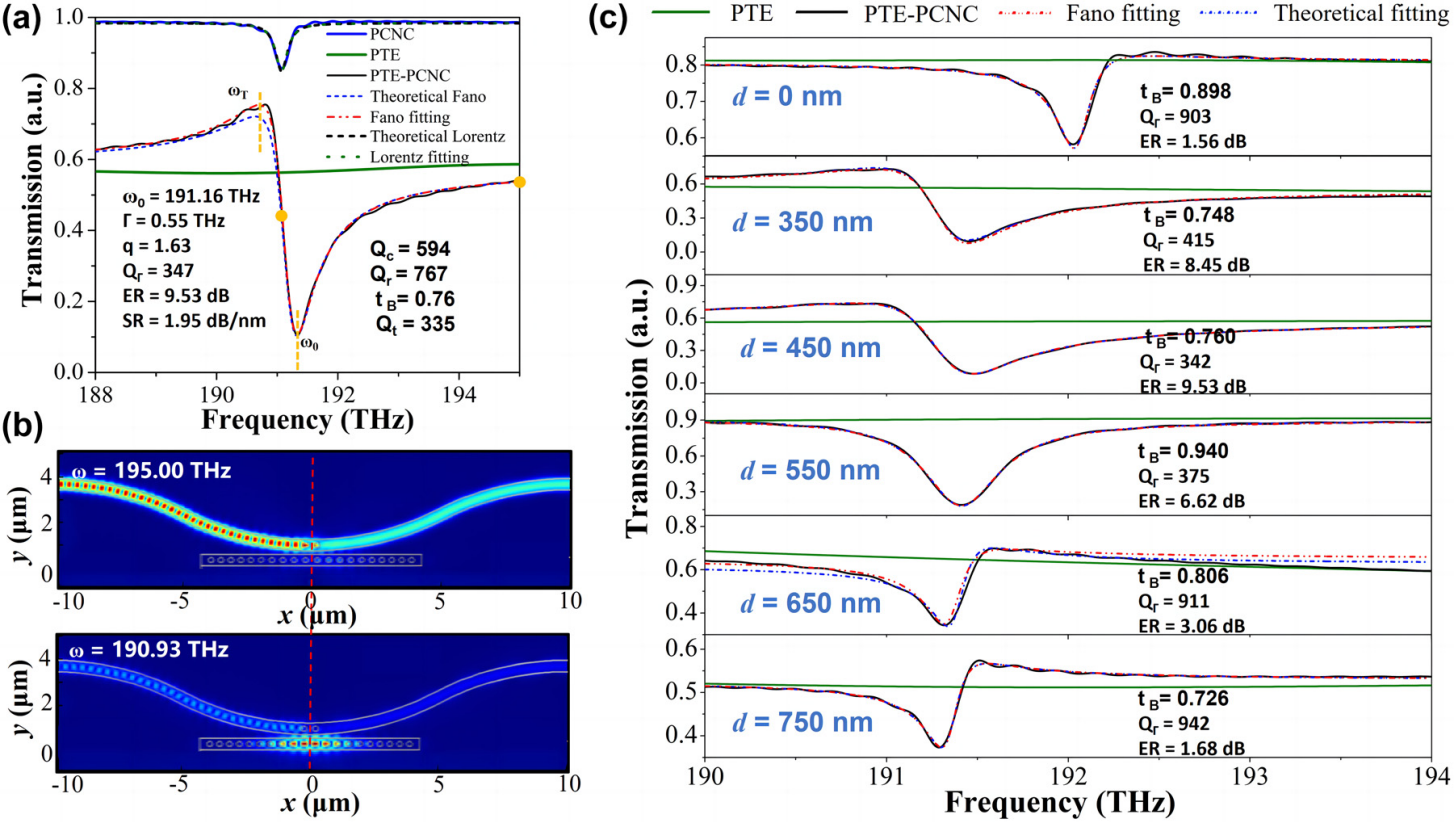
Simulation results. (a) The simulated transmission spectra of the ordinary side-coupled PCNC without PTE, the line-defect waveguide with PTE, the proposed side-coupled PTE-PCNC, and the corresponding theoretical transmission, Fano fitting, and Lorentz fitting. (b) The electric field distribution in the middle plain of the Fano mode PCNC at frequency of 195.00 THz and 190.93 THz. Symmetry plane is indicated by the red dashed line. (c) The transmission spectra of the PTE-PCNC with different PTE lengths.

Taking *N*_taper_ = 6 and *g* = 200 nm for example, the numerical and theoretical transmission spectra are shown in Figure 2(a). To achieve the value of *t*_*B*_ and make a comparison, the transmission spectra of the line-defect waveguide with PTE and the ordinary side-coupled cavity without the PTE are also shown. As shown, when there is no PTE, transmission line-shape with Lorentzian symmetric is achieved. According to the Lorentz fitting and the transmission of the ordinary side-coupled cavity [44], the *Q*_*t*_, *Q*_*r,*_ and *Q*_c_ are 796, 859, and 10,886, respectively. The ER (∼0.63 dB) is rather low due to the under-coupled condition (*Q*_*c*_ > *Q*_*r*_).

While, for PTE-PCNC, a sharper and higher-ER asymmetric Fano line-shape appears in the transmission spectrum, though higher insertion loss ∼2.22 dB is generated due to the PTE. According to the transmission spectrum of line-defect waveguide with PTE, the transmission power coefficient *t*_*B*_ of PTE is ∼0.76 at the resonance frequency of ∼191.16 THz (∼1569 nm). The resonance frequency is close to but slightly larger than the air-band edge (∼186.9 THz, red circle) of the central mirror segment obtained from the band-diagram shown in Figure 1(b). This can be attributed to the quadratically decreased lattice constant of the structure away from the center, which results in decreased overlap between the cavity mode and dielectric material. The frequency offset, which can be estimated using perturbation theory, decreases as the number of modulated mirror segments *N*_taper_ increases. The corresponding calculated electric field (*E*) distributions of PTE-PCNC in the *xy* plane (*z* = 0) for the modes at frequency of 190.93 THz and 195.00 THz are shown in Figure 2(b). When the waveguide mode is non-resonant with the cavity mode, the light cannot be coupled into the cavity and is reflected mostly by the PTE. While, the light with the cavity resonance frequency couples into the cavity and transmits to the output port of the waveguide. Therefore, Fano type peak appears in the transmission spectrum.

The total *Q*-factor of the cavity can be evaluated from parameters obtained by fitting the simulated transmission spectrum to a Fano line-shape formula [45]:

(6) $$T\left(\omega \right)=T_{0}+A_{0}\frac{{\left[q+2\left(\omega -\omega_{0}\right)/\Gamma \right]}^{2}}{1+{\left[2\left(\omega -\omega_{0}\right)/\Gamma \right]}^{2}}$$

where *q* is the Fano asymmetry parameter, *ω*_0_ is the resonance frequency of the cavity mode, Γ is the resonance linewidth (denoted by frequency difference at the transmission peak and dip *ω*_0_ − *ω*_T_), *T*_0_ and *A*_0_ are constant offset and scaling factors, respectively. Such a fit is shown in Figure 2(a), using Eq. (6) with fit parameters *T*_0_ = 0.75, *A*_0_ = −0.17, *q* = 1.63, *ω*_0_ = 191.16 THz, and Γ = 0.55 THz. The simulated *Q*-factor is defined by the ratio between *ω*_0_ and Γ (*Q*_Γ_ = *ω*_0_/Γ). Therefore, Fano resonance with an asymmetric line-shape with a *Q*_Γ_ of 347 and an ER of 9.53 dB is achieved. Additionally, according to Eq. (5), with known calculated values *t*_*B*_ (0.76), *Q*_*c*_ (568), and *Q*_*r*_ (984), for *P* = −1, the calculated theoretical transmission spectrum is quite consistent with the simulated transmission spectrum shown in Figure 2(a), which shows the applicability and accuracy of the theoretical model and analysis process. The corresponding calculated total *Q*-factor *Q*_*t*_ (*=*(1*/Q*_*r*_ *+* 1*/Q*_*c*_)^−1^) is ∼360, which is mainly limited by *Q*_*c*_. Moreover, from the calculated distribution of the resonance mode, ∼24 % of electric field energy is located in low refractive index region calculated by

(7) $$f=\frac{\underset{\varepsilon_{\text{low}}}{\int }\text{d}V\left(\varepsilon •{\left|E\right|}^{2}\right)}{\int \text{d}V\left(\varepsilon •{\left|E\right|}^{2}\right)}$$

To investigate the influence of the two-hole-assisted PTE to the line-shape of Fano resonance, the transmission spectra of the device with different PTE lengths *d* are simulated as shown in Figure 2(c). As shown, the features of the Fano resonance can be simply controlled via the amplitude transmission coefficient of the PTE. The value *t*_*B*_ changes with *d* due to the phase shift caused by the optical length difference between the PTE holes acting like a Fabry–Perot (FP) cavity. As shown in Figure 2(c), the red parity is observed when *d* = 0 nm, 650 nm, and 750 nm. And the blue parity is observed when *d* = 350 nm, 450 nm, and 550 nm. In addition, the design of PTE also has an effect on the coupling coefficient between PCNC and PTE, resulting in the change of ER and total *Q*-factor. For *d* = 0 nm, 650 nm, and 750 nm, to improve the ER, the coupling gap should be decreased to change the under-coupled condition to critically-coupled condition. But it is hard to be fabricated due to the limitation of critical dimension. For the two-hole-assisted PTE model with *d* = 450 nm, the ER is higher than the other designs shown in Figure 2(c). Considering the fabrication limitation and the device performance, the two-hole-assisted PTE with distance around 450 nm is preferred for our design. Therefore, for our fabricated devices, the distance *d* is set as 448 nm, which is the same as the lattice constant of the central tapered segment in PCNC.

**Figure 3:**
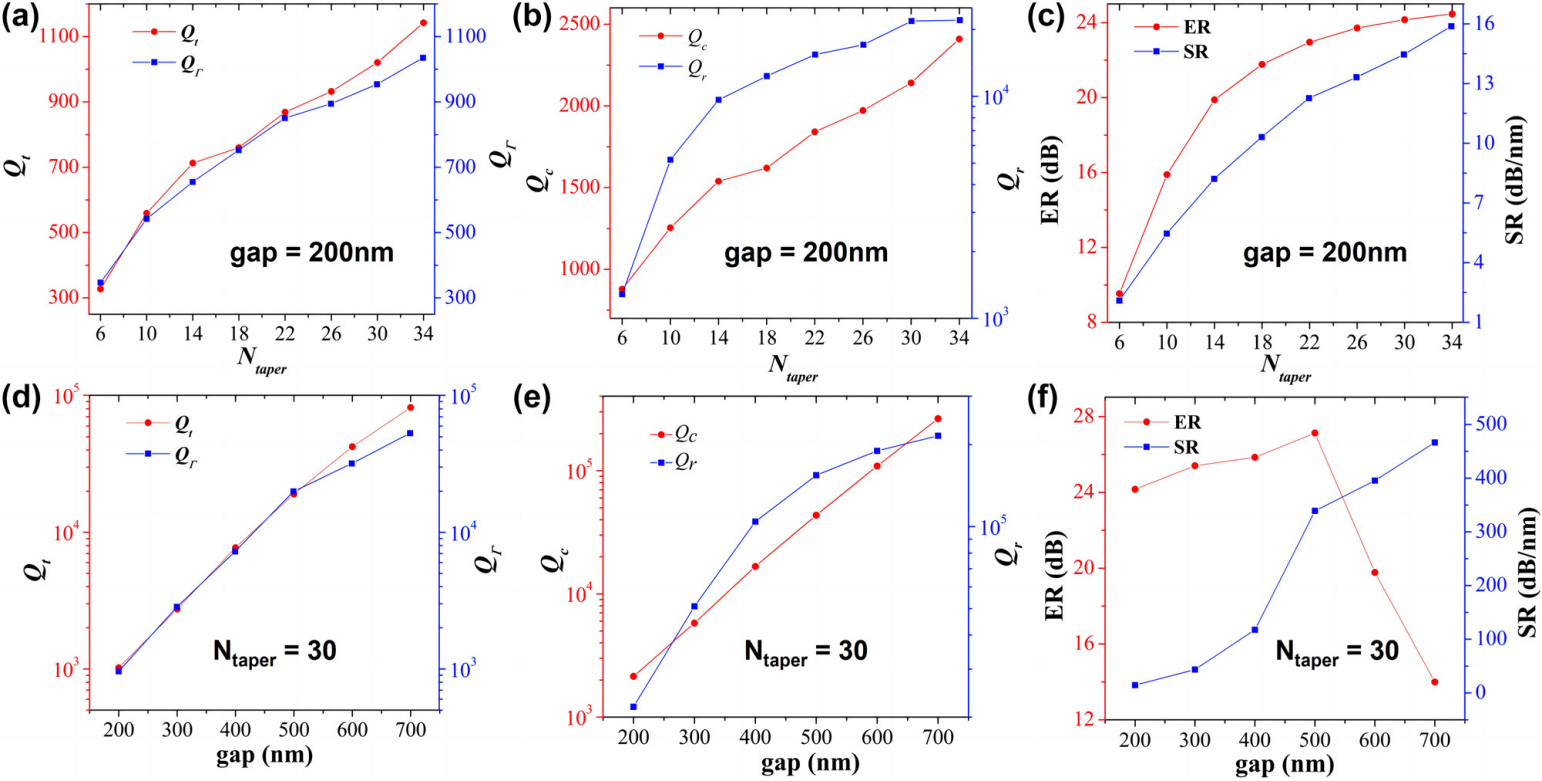
Calculated (a) *Q*_*t*_, *Q*_Γ_, (b) *Q*_*c*_, *Q*_*r*_, and (c) ER and SR of the devices with different taper holes number *N*_taper_ for *g* = 200 nm. Calculated (d) *Q*_*t*_, *Q*_Γ_, (e) *Q*_*c*_, *Q*_*r*_, and (f) ER and SR of the devices with different coupling gap width for *N*_taper_ = 30.

To further optimize the transmission spectrum of Fano asymmetric line-shape in PTE-PCNC for the realization of high *Q*_*t*_ and high ER, according to Eq. (5), the radiation *Q*_*r*_ and coupling *Q*_*c*_ of the device should be improved by investigating the two key parameters *N*_taper_ and *g*. Figure 3 shows the optimization process for the two geometric parameters.

Firstly, the PTE-PCNC with different *N*_taper_ is simulated using 3D-FDTD method, while keeping other parameters unchanged. The corresponding calculated *Q*_*t*_, *Q*_Γ_, *Q*_*c*_, *Q*_*r*_, ER, and slope rate (SR) of devices are given in Figure 3(a)–(c). With the change of *N*_taper_, the discrete states (i.e., resonant frequency *ω*_0_ of PCNC) are changed. Therefore, the coupling condition would be changed, leading to the variety in the Fano resonance. As shown, the calculated *Q*_*t*_, *Q*_*c,*_ and *Q*_*r*_ increase by adding the number of holes *N*_taper_. And the *Q*_*t*_ calculated from cavity loss corresponds well with the *Q*_Γ_ calculated from the transmission spectrum, due to the relatively low *Q*-factor. Here, according to the calculated values of *Q*_*c*_ and *Q*_*r*_ shown in Figure 3(b), the total *Q*-factor is limited by *Q*_*c*_, which can be improved by increasing the coupling gap width *g*. Furthermore, the extracted ER from the transmission spectra tends to be saturated as *N*_taper_ increases to 30, as shown in Figure 3(c).

Secondly, for *N*_taper_ = 30, *Q*-factors and ERs of PTE-PCNC with different *g* are calculated to further investigate the influence of *g* on Fano resonance line-shape. As shown in Figure 3(d), the *Q*-factor *Q*_*t*_ increases exponentially with the increase of *g*, until it reaches about 9.1 × 10^4^ (limited by *Q*_*r*_) for *g* equaling to 700 nm. The reason is that *Q*_*c*_ increases exponentially with *g*, whereas *Q*_*r*_ increases slowly when *g* > 500 nm, as shown in Figure 3(e). Additionally, when *g* is large than 500 nm, the *Q*_Γ_ calculated from the transmission spectrum is much less than the *Q*_*t*_ calculated from cavity loss. This can be attributed to the very large photon life time of our ultra-high *Q*-factor when *g* > 500 nm. It becomes nearly impossible to model transmission using the 3D-FDTD method directly when the total *Q*-factor is too high. And the increase in the coupling gap *g* can reduce the coupling coefficient and causes the PTE-PCNC system to be tuned gradually from the initial over-coupled condition to critically-coupled and further to under-coupled conditions. Therefore, it is observed in Figure 3(f) that, as the increase of *g*, ER increases when *g* is less than 500 nm, and then decreases dramatically afterwards. For *g* = 500 nm and *N*_taper_ = 30, high *Q*-factor of 1.9 × 10^4^, and ER of 27 dB are obtained at wavelength ∼1594 nm, resulting in a high SR ∼339 dB/nm. Although SR is not very high in this case, the value of more than 300 dB/nm is still achieved, thus allowing the realization of high-performance sensors and switches. Note that in further study the total *Q*-factor can be improved by modifying the structural parameters (such as wider waveguide width and larger hole radius) of PCNC to achieve higher *Q*_*r*_ (>10^7^).

## 4 Fabrication and characterization

### 4.1 Fabrication

Our devices were fabricated on an 8-inch SOI wafer with a 220 nm-thick top-silicon layer and a 3 µm-thick BOX in a CMOS pilot line. To realize optical characterization, the waveguides were integrated with TE grating couplers. The DUV photolithography was employed to form the devices patterns on photoresist as a soft mask. Double Inductively coupled plasma (ICP) etching processes were applied to transfer the patterns from the photoresist layer to silicon to form waveguides and devices. 220 nm full etching was defined for waveguides and circle holes, and 70 nm shallow etching was defined for grating lines. The devices were oxide-embedded through oxide deposition by plasma-enhanced chemical vapor deposition (PECVD) and planarization down to the top of the silicon layer.

**Figure 4:**
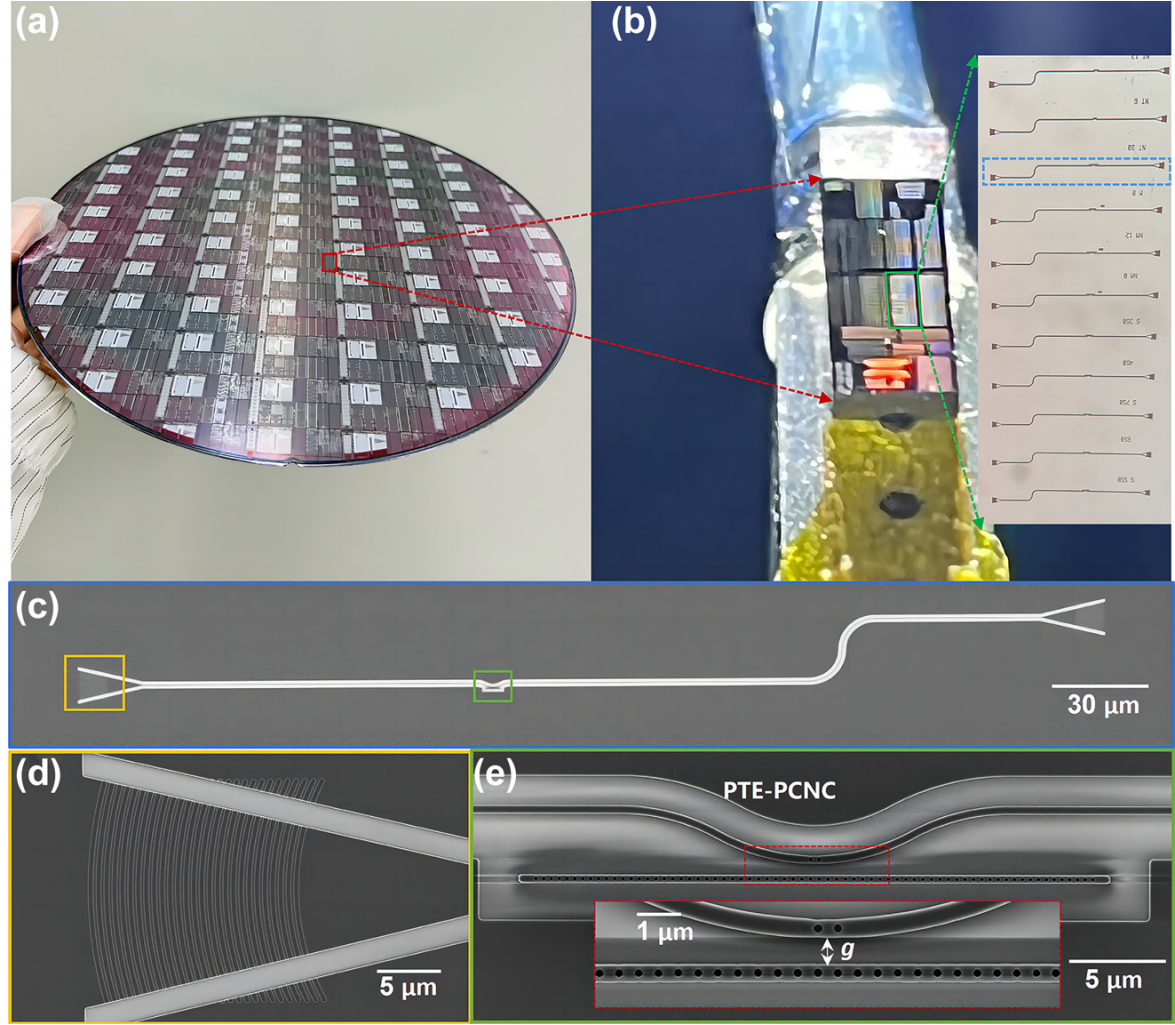
Fabrication results. (a) Photography of the finished 8-inch wafer fabricated by MPW run in a CMOS pilot line. (b) Image of a single die from the 8-inch wafer. The inset microscope image shows the fabricated devices. The SEM images of fabricated (c) device, (d) grating coupler, and (e) side-coupled PCNC with PTE fabricated at ∼180 nm technology node.

Figure 4(a) shows the finished 8-inch wafer fabricated by MPW run in a CMOS pilot line at an ∼180 nm technology node. Figure 4(b) shows the single die from the 8-inch wafer. And the inset microscope image shows the fabricated devices with different *N*_taper_ and *g*. Figure 4 (c) shows the scanning electron microscope (SEM) image (*xy* plane) of the fabricated device, in which light is coupled into the bus waveguide by the focusing grating coupler, then passes through the PTE-PCNC, and finally gets directed out by the other grating coupler. The SEM images of the fabricated grating coupler and PTE-PCNC are shown in Figure 4(d) and (e), respectively. A close-up SEM image of the evanescent coupling region between PCNC and coupling defect waveguide is shown in the zoom-in picture of Figure 4(e), indicating well-defined circular holes and the coupling region. The fabricated devices were characterized by measuring the transmission, using a tunable laser (from 1500 to 1620 nm), and cleaved single-mode fibers for input/output coupling. In the following experimental results, the shown measured transmission spectra were obtained by normalizing the spectra to a reference waveguide.

### 4.2 Measurement and analysis

**Figure 5:**
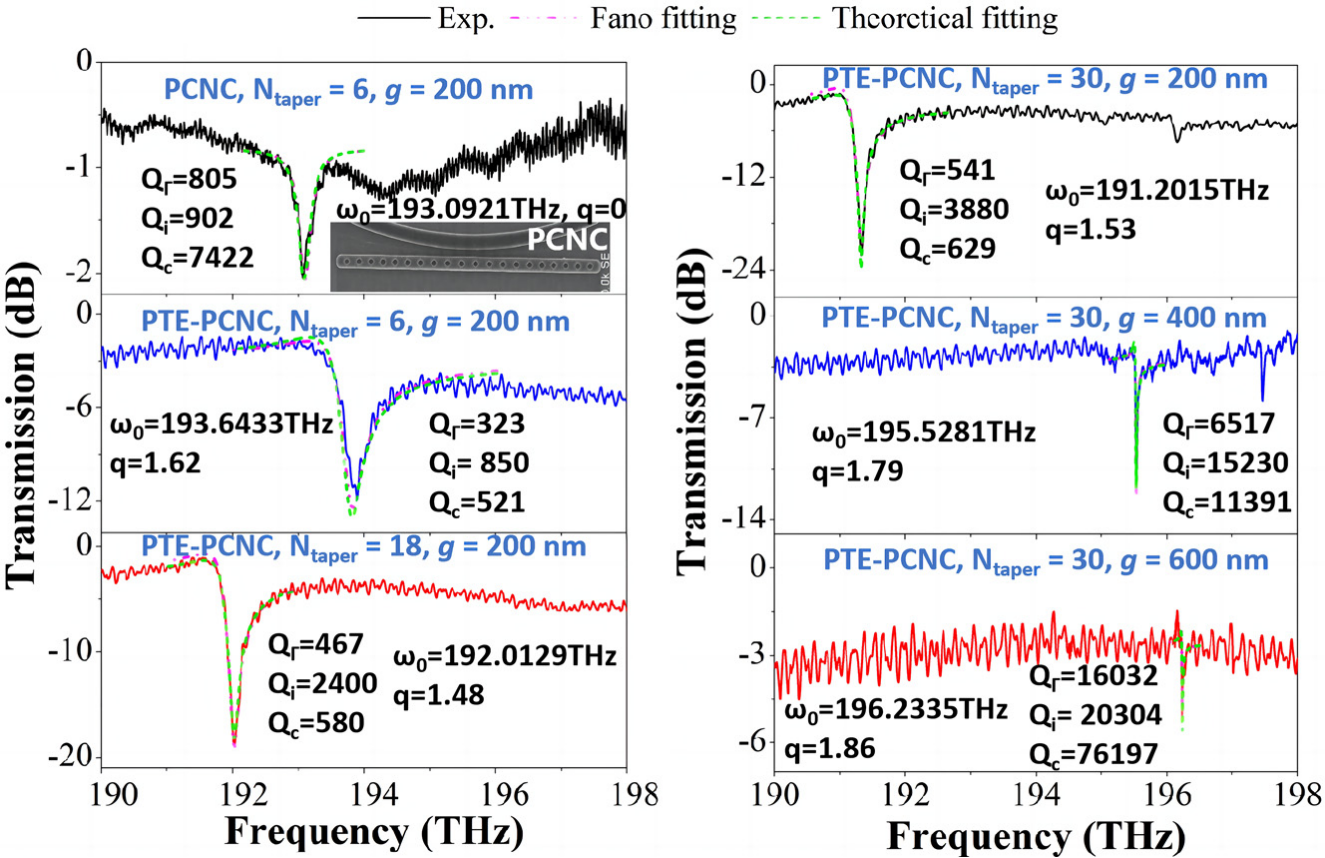
Measured and fitted transmittance spectra for the fundamental modes of the normal side-coupled PCNC and PTE-PCNCs with *w* = 450 nm, *r* = 90 nm, *a*_center_ = 448 nm, and *a*_end_ = 480 nm at various *g* and *N*_taper_.

To demonstrate the characteristics of the proposed Fano resonance from air-mode PTE-PCNC, the normal side-coupled air-mode PCNC with *N*_taper_ = 6 (*g* = 200 nm), the PTE-PCNCs with *g* = 200 nm (*N*_taper_ = 6, 18 and 30) and *N*_taper_ = 30 (*g* = 400, 600 nm) are fabricated and measured. The normalized experimental transmission spectra are shown in Figure 5. And the extracted resonance frequency *ω*_0_, asymmetry parameter *q*, total *Q*-factor *Q*_*Γ*_, intrinsic *Q*-factor *Q*_*i,*_ and coupling *Q*-factor *Q*_c_ are marked in Figure 5 correspondingly. For the fabricated device, *Q*_*i*_ is composed of *Q*_*r*_ and *Q*_*s*_.

As shown, for normal side-coupled PCNC with *N*_taper_ = 6 and *g* = 200 nm, the measured transmission has Lorentzian resonance line-shape (*q* = 0) at resonance frequency of 193.0921 THz. The small frequency mismatch between the simulation and the experiment is possibly caused by fabrication imperfections or simulation parameter settings, such as mesh accuracy. The insertion loss is ∼0.5 dB at the non-resonance frequency. A Lorentzian fit to the measured transmission yields a total *Q*-factor of 805, which for the measured ER of 1.2 dB corresponds to an intrinsic *Q*-factor of *Q*_*i*_ = 902 and a coupling *Q*-factor of *Q*_*c*_ = 7422. Compared with the simulation results shown in Figure 2(a), the smaller measured *Q*_*c*_ may be caused by the shallow etching depth in the coupling region due to the RIE-lag effect when *g* = 200 nm.

As expected, for PTE-PCNC with *N*_taper_ = 6 and *g* = 200 nm, a sharper and higher-ER asymmetric Fano line-shape appears in the measured transmission spectrum, though the total *Q-*factor *Q*_Γ_ is 323 achieved by fitting the transmission spectra using the Fano line-shape formula. The asymmetric parameter *q* is estimated as *q* = 1.62, proving the consistency of the simulation result. The insertion loss is ∼2.5 dB at the non-resonance frequency. Inserting the data extracted from the spectra into Eq. (5) and 1/*Q*_Γ_ = 1/*Q*_*i*_ + 1/*Q*_*c*_, the experimental *Q*_*i*_ = 850 and *Q*_*c*_ = 521 are derived. As *N*_taper_ grows from 6 to 30, the resonance frequency of the fundamental mode decreases from 193.6433 THz to 191.2015 THz, and the corresponding total *Q*-factor *Q*_*Γ*_ increases from 323 to 541. Though both derived *Q*_c_ and *Q*_*i*_ increase with the growth of *N*_taper_, the results are much smaller than the theoretical ones shown in Figure 3(b). When *N*_taper_ = 30 and *g* = 200 nm, the derived experimental *Q*_*i*_ and *Q*_*c*_ are 3880 and 629, respectively, resulting a high ER ∼21.5 dB and SR ∼7.3 dB/nm. Since the simulated *Q*_*r*_ is larger than 2 × 10^4^, the *Q*_*i*_ is mainly limited by fabrication imperfection induced scattering loss *Q*_*s*_.

By increasing the coupling gap from 200 nm to 600 nm with a step of 200 nm, the experimental coupling condition changes from initial over-coupling (*Q*_*c*_ < *Q*_*i*_) to under-coupling (*Q*_*c*_ > *Q*_*i*_). The increase tendency of the experimental results (*Q*_Γ_, *Q*_*c*_ and *Q*_*i*_) are in agreement with the simulation results shown in Figure 3. And *Q*_*c*_ grows more rapidly than *Q*_*i*_, resulting the monotonically decreasing of ER. When *g* = 400 nm, the fundamental mode is achieved at resonance frequency of 195.5281 THz. The corresponding *Q*_Γ_, ER, and SR are 6.52 × 10^3^, 10.8 dB, and 53 dB/nm, respectively. The derived *Q*_*i*_ and *Q*_*c*_ are 1.52 × 10^4^ and 1.34 × 10^4^, respectively. Increasing the gap distance to 600 nm, the derived *Q*_*i*_ grows weakly to 2.03 × 10^4^, while *Q*_*c*_ increases rapidly to 7.62 × 10^4^, resulting in a lower ER ∼ 2.91 dB and SR ∼ 49 dB/nm. The phenomenon demonstrates that the measured total *Q*-factor 1.60 × 10^4^ is mainly limited by the fabrication induced scattering loss since the calculated *Q*_*r*_ is ∼2 × 10^5^. Therefore, in further studies, how to decrease the fabrication induced imperfection in CMOS line should be studied to achieve higher *Q* and ER.

In addition, as observed, higher-order resonances modes can be observed when *N*_taper_ = 30. Compared with the fundamental mode, higher-order modes are significantly suppressed, due to the spatial separation of the cavity and waveguide modes. For *g* = 400 nm, higher-order resonance mode at frequency of 197. 4576 THz is present with higher total *Q*-factors of 6.57 × 10^3^. However, the ER is only ∼3.9 dB, and it disappears at larger gaps. This feature enables single-frequency operation over a very broad frequency range, compared to ring resonators.

**Figure 6:**
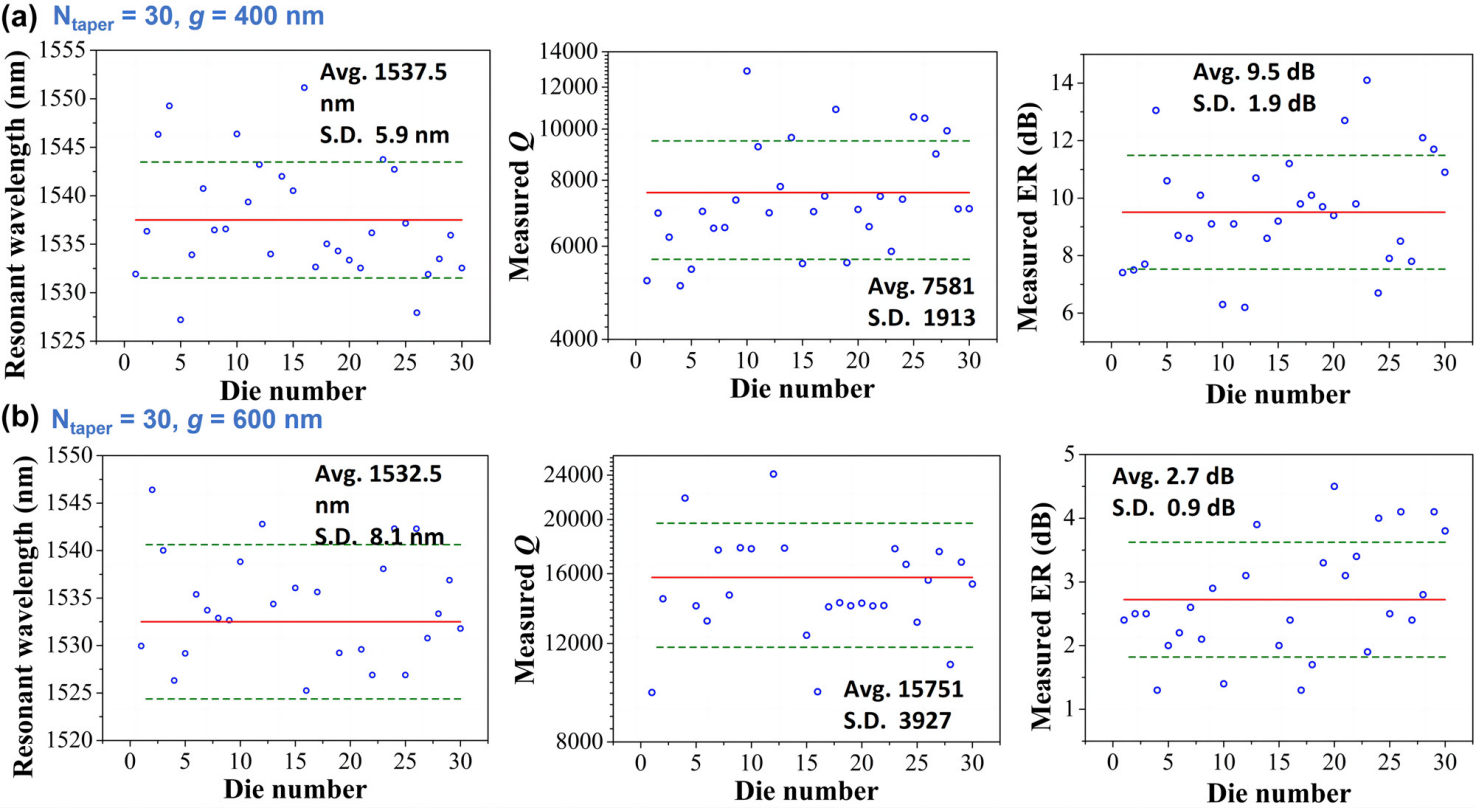
Experimental resonant wavelengths, *Q* and ER distributions of 30 measured dies for PTE-PCNCs with *N*_taper_ = 30, (a) *g* = 400 nm and (b) *g* = 600 nm, respectively. The solid line indicates the average values. The dashed line indicates the standard deviation values.

To achieve more insightful information than single device, a sufficiently large amount (30 dies) of PTE-PCNCs with *N*_taper_ = 30 is tested. The variations of the measured resonance wavelengths, *Q*-factors, and ERs derived from the measured transmission spectra for *g* = 400 nm and 600 nm are shown in Figure 6(a) and (b), respectively. Fluctuations in the measured values are due to intrinsic disorder introduced by the fabrication process. The average resonance wavelength values for *g* = 400 nm and 600 nm are 1537.5 nm and 1532.5 nm, respectively. The result reveals red-shift of resonance due to increased effective refractive index as the coupling gap *g* narrows. The standard deviations are 5.9 nm and 8.1 nm, respectively, which should be decreased in further study by improving the structure fabrication tolerance. For *g* = 400 nm and 600 nm, the highest *Q*-factors are 1.29 × 10^4^ and 2.41 × 10^4^, which correspond to ERs of 6.3 dB and 3.1 dB, respectively. In average, the *Q*-factors of the fundamental mode for *g* = 400 nm and 600 nm are about 7.58 × 10^3^ and 1.58 × 10^4^, respectively, for 30 measured resonances in total. Thus, here a large *Q* factor has been achieved systematically. These results indicate that the mass manufacture of high-*Q* Fano resonance from air-mode PTE-PCNC is feasible by using CMOS-compatible technologies.

**Figure 7:**
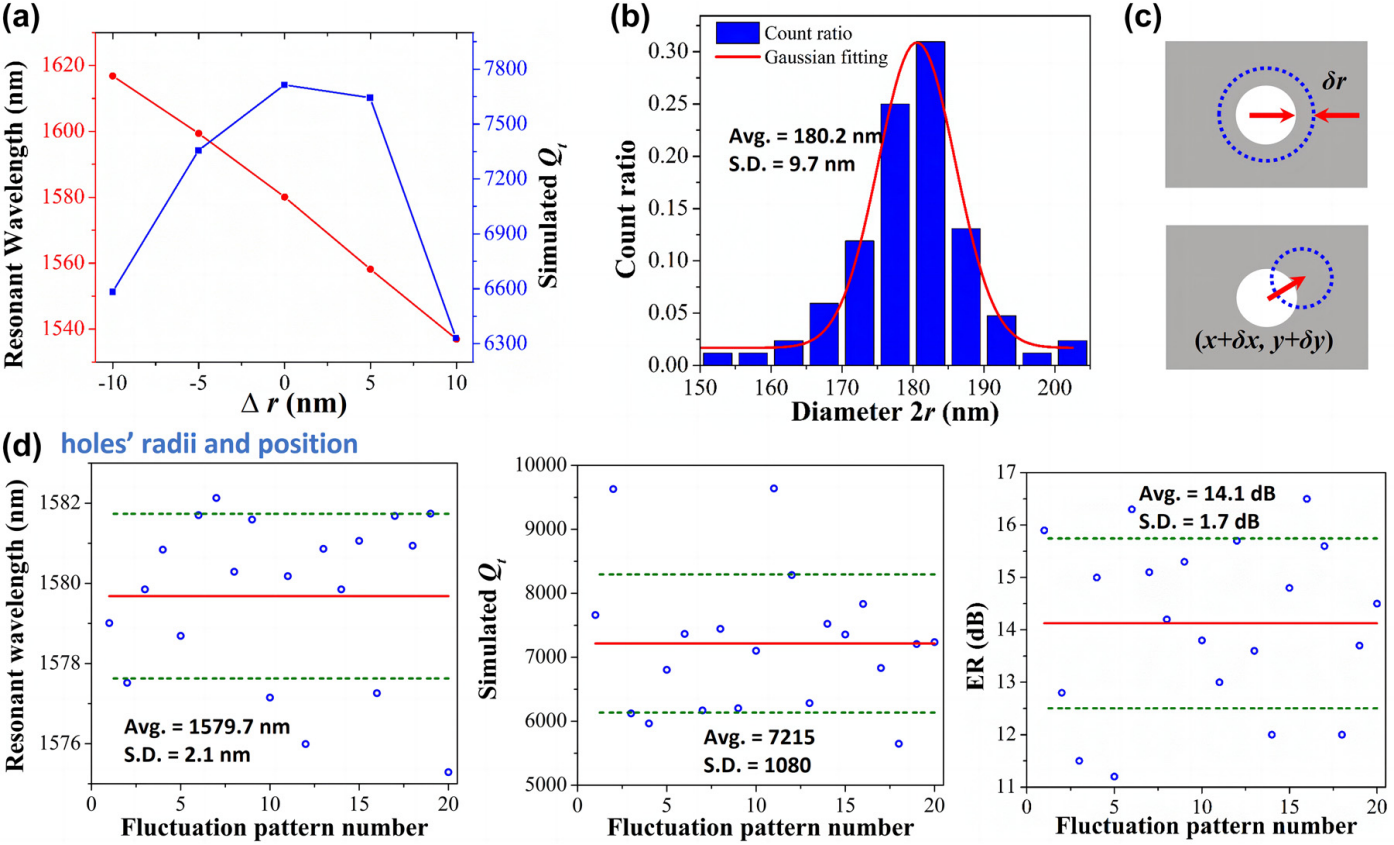
Fabrication tolerance analysis. (a) The variation of resonance wavelength and total *Q*-factor versus uniform hole radius offset ∆*r*. (b) The statistical distribution of the air holes’ diameters exhibiting Gaussian distributions. (c) Holes’ radii and positions perturbed by random deviations *δ*_*r*_, *δ*_*x,*_ and *δ*_*y*_. (d) The corresponding fluctuation of resonance wavelength, total *Q*-factor and ER for 20 different random patterns.

To better understand the origin of the statistical variations of the device characteristics, we numerically estimate the effects of fabrication errors including fluctuation of air–hole radii, positions, waveguide width and coupling gap. Taking PTE-PCNC with *N*_taper_ = 30, *g* = 400 nm for example, to study the fabrication error tolerance of the cavity, firstly, the influence of fabrication offsets in the air hole radius with uniform offset (∆*r*) is studied. We set the radius offset varying from −10 nm to 10 nm with a step of 5 nm. As shown in Figure 7(a), with the increase of holes’ radius, the resonance wavelength shifts to shorter wavelength range due to the decrease of effective refractive index. And the total *Q*-factor decreases with the increase of offset. When the offset is ±10 nm, the total *Q*-factor of above 6332 is achievable. However, in practice, the radius offset for each holes on the device is not uniform. To study the fabrication error in more detail, from SEM images of the devices on the die from center of the wafer, the distribution of air hole diameters can be described by a Gaussian distribution with mean value being 180.2 nm and standard deviation of 9.7 nm, as shown in Figure 7(b). Since the positions of the air holes are hard to determine accurately due to the quadratically tapered lattice constant structure, we also assume the maximum deviation of positions to be same as the maximum deviation of radii. Therefore, 3D-FDTD calculations taking the randomness of the hole radii and positions into account are performed. As shown in Figure 7(c), the holes’ radii and positions are perturbed by random deviations *δr*, *δ*_*x,*_ and *δ*_*y*_ varying from −5 nm to 5 nm. Figure 7(d) shows 20 different random results. The average resonant wavelength, *Q*-factor, and ER are 1579.7 nm, 7215, and 14.1 dB, respectively. And the corresponding standard deviations are 2.1 nm, 1080, and 1.7 dB, respectively. The average *Q*-factor corresponds well with the measured characteristics of the devices shown in Figure 7(a). Note that for different dies the mean value of the holes’ diameters has slightly shift, therefore the simulated standard deviations here are smaller than the measured results.

**Figure 8:**
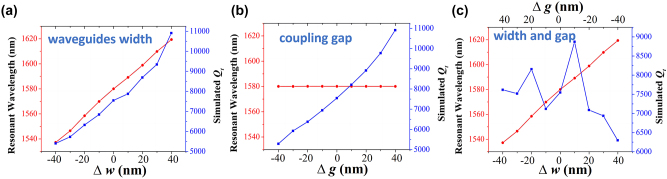
The variation of resonance wavelength and total *Q*-factor versus (a) waveguide width offset ∆*w*, (b) coupling gap offset ∆*g,* and (c) ∆*w* and ∆*g* changing together.

Since the measured average resonance wavelength of the devices shift to 1537.5 nm, we study the effect of the fabrication variation of waveguide width and coupling gap of the PTE-PCNC structure on the performance of the resonance mode, as presented in Figure 8. Because the fabrication variation of waveguide width generally fluctuates within 10 %, therefore, we set the waveguide width offset (∆*w*) varying from −40 nm to 40 nm with a step of 10 nm. As shown in Figure 8(a), with the increase of the waveguide width, both the resonance wavelength and *Q*-factor increase due to the increased effective refractive index and stronger light confinement. Keeping the waveguide width 450 nm unchanged, setting the coupling offset (∆*g*) varying from −40 nm to 40 nm as shown in Figure 8(b), the resonance wavelength almost keep unchanged, while the *Q*-factor increases with the decreased coupling gap due to the decreased coupling loss. In practice, as the waveguide width decreased, the coupling gap correspondingly became larger. Therefore, as shown in Figure 8(c), when ∆*w* and ∆*g* working together, the total *Q*-factor of 6258–8935 is achievable. When waveguide width and coupling gap equal to 410 nm and 440 nm, respectively, the corresponding resonant wavelength, *Q*-factor are 1539 nm and 8115, respectively. This, together with the results shown in Figure 7 for tuning of the holes’ size and position, demonstrates the good correspondence between the measured and simulated operating wavelength, *Q*-factor and ER taking into account a reduction of 40 nm in width, increase of 40 nm in coupling gap, 5 nm random fluctuation in holes’ radii and position. This in turn demonstrates the accuracy and reliability of CMOS technology for the fabrication of the proposed PTE-PCNC structure. Therefore, the structure retains good performance even after several nanometer fabrication fluctuations. Note that for PTE-PCNC structure with larger coupling gap, the waveguide and gap offsets caused by fabrication are larger, resulting in the red-shift of the measurement average resonance wavelength when *g* = 600 nm as shown in Figure 6. Though the value of random deviations *δr*, *δ*_*x,*_ and *δ*_*y*_ are a few times larger than the value obtained with EBL where standard deviation of hole diameters less than 1 nm has been reported [46, 47], the performance of our device exceeds previous reported air-mode PCNC [48] and Fano resonance from PCNC [17, 21, 28] fabricated by EBL.

## 5 Discussion

**Figure 9:**
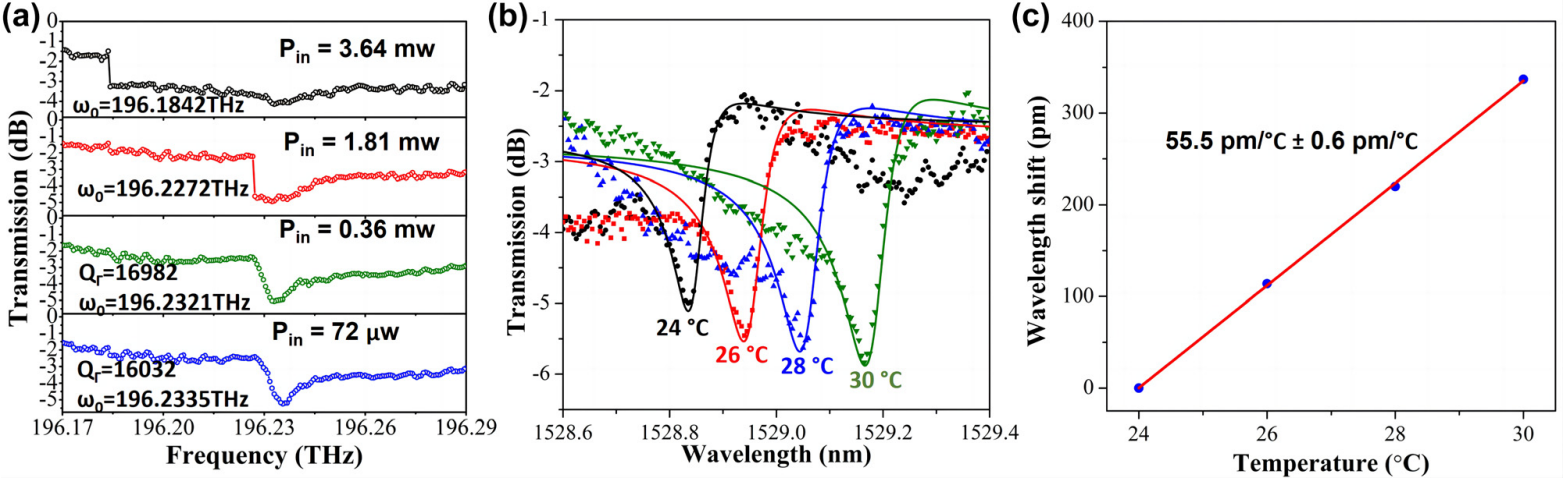
Nonlinear and thermal tuning characterization. (a) Measured transmission spectra of PTE-PCNC with *N*_taper_ = 30 and *g* = 600 nm at different input powers *P*_in_. (b) Measured resonant spectra as a function of the applied temperature with *P*_in_ = 72 μw. (c) The corresponding measured resonant wavelength shift as a function of temperature.

We expect that the mass manufacture of our proposed Fano resonance from PTE-PCNC for applications such as optical switching, sensing, filtering or modulating would be possible. For our proposed PTE-PCNC, experimental low, moderate and high *Q*-factors ranging from ∼500 to ∼1.6 × 10^4^ have been demonstrated to meet different application requirements. Then, we simply discuss the nonlinear and thermal tuning characterization of the fabricated PTE-PCNC with *N*_taper_ = 30 and *g* = 600 nm. Figure 9(a) shows the output spectrum from the cavity which has *Q*-factor of 1.60 × 10^4^ at different coupled in laser powers *P*_in_ (72 μw, 0.36 mw, 1.81 mw, and 3.64 mw). *P*_in_ are deduced by subtracting input grating coupler loss from the launched power 0.2, 1.0, 5.0, and 10.0 mw, respectively. The coupling efficiency of the fiber-to-grating is ∼4.4 dB at wavelength of 1529 nm using a coupling angle of 12° off the vertical. With the increase of the input power, the center wavelength has a red-shift, which is caused by the thermo-optic effect. The overall resonance wavelength shift is ∼38 pm. In addition, a sharp increase of the line shape is observed when the input power increases to 1.81 mw, indicating a strong evidence for optical bi-stability. This is due to the combined effect of the thermal refractive index-change and Kerr nonlinear index change as the laser scan wavelength is red detuned [16]. The bi-stable threshold is among 0.36–1.81 mw.

To further investigate the thermal tuning ability of the fabricated PTE-PCNC, a temperature controller platform is used to control the temperature of the device. The power of the laser is set as 0.2 mw. Figure 9(b) shows the measured normalized transmission spectra of the device with the ambient temperature changing from 24 °C to 30 °C with a step of 2 °C. As expected, as the temperature increased, the transmission dips shift to longer wavelengths due to the increase of the core refractive index of the Si waveguide. Figure 9(c) shows that the wavelength shift induced by temperature is linear, indicating a temperature sensitivity of ∼55.5 pm/°C. The temperature sensitivity is comparable with previous reported PCNC structures [48, 49]. In further fabrication, on top of the upper SiO_2_ cladding layer, titanium nitride (TiN) micro-heaters can be integrated with the PTE-PCNC devices to realize thermo-optic tuning. And the add-drop structure can also be further designed by adding bus waveguide on the other side of the PCNC.

Since the nonlinear phenomenon has been demonstrated for high *Q*-factor (>10^4^) PTE-PCNC at low threshold power, it has potential for ultrafast all-optical modulation and switching [16]. Based on the thermal tuning effect, considering the trade-offs in speed and energy cost, PTE-PCNC structures with moderate *Q*-factor (<10^4^), low insertion loss (∼2.5 dB), and high ER (>15 dB) are desirable for applications such as filters [10, 11, 49], switches [7–9, 17, 18, 50], and spectrometers [12, 20, 51]. For example, a side-coupled FSR-free resonance with 3 dB bandwidth of 0.25 nm and *Q* factor of 5942 has been demonstrated using as a filter [49]. A PCNC-based thermo-optic Mach–Zehnder interferometer switch has been demonstrated using resonance with 3 dB bandwidth 0.68 nm (i.e., 85 GHz, *Q*-factor of 2300) and ER ∼ 15 dB [50]. Tunable Fano-enhanced nanobeams with moderate *Q*-factor (4319), high ER (>26 dB), and low insertion loss (4.7 dB) have been used as functional components for on-chip spectrometer [20].

In addition, the device with oxide cladding opening window can be used for biochemical sensing, achieving simulated refractive index sensitivity of ∼225 nm/RIU. The value is higher than most reported dielectric mode PCNCs for sensing [5, 6, 48]. And in further, the *Q*-factor of the device with liquid cladding can be optimized to 10^4^, which is high enough to allow for a high sensing resolution. Additionally, the footprint of the device is only 1 µm × 25 µm. Due to the side coupling waveguide and SiO_2_ cladding, the designed devices can be directly used as functional components and integrated with other on-chip devices in future practical applications.

## 6 Conclusions

In conclusion, we have demonstrated an ultra-compact air-mode PTE-PCNC structure in SOI platform to generate Fano resonance with high *Q*-factor suitable for DUV lithography. The TCMT and 3D-FDTD method are utilized to analyze the designed structure, the Fano resonance lineshapes and the corresponding partial *Q*-factors. Experimentally average high *Q*-factor of ∼1.58 × 10^4^ is achieved, which is mainly limited by imperfection induced scattering loss. The average insertion loss of the device is ∼2.5 dB. And the characterization of thermo-optic bi-stability and thermal tuning of the device is performed to explore its application. The results and analysis will contribute to building ultra-compact lab-on-chip resonance-based photonic components such as sensors, switches, filters, reflectors, and so on.
